# Synthesis of 2-Substitued Indoles via Pd-Catalysed Cyclization in an Aqueous Micellar Medium

**DOI:** 10.3390/molecules26133917

**Published:** 2021-06-26

**Authors:** Sofia Siciliano, Elena Cini, Maurizio Taddei, Giorgia Vinciarelli

**Affiliations:** Dipartimento di Biotecnologie, Chimica e Farmacia, Università degli Studi di Siena, Via A. Moro 2, 53100 Siena, Italy; sofia.siciliano@student.unisi.it (S.S.); elena.cini@unisi.it (E.C.); giorgia.vinciarel@student.unisi.it (G.V.)

**Keywords:** microwaves, homogeneous catalysis, heterocycles

## Abstract

The synthesis of 2-substituted indoles starting from the corresponding unprotected 2-alkynylanilines was made possible in 3% TPGS-750-M water using Pd(OAc)_2_ alone as the catalyst. The reaction was sensitive to the heating mode respect to the nature of the starting material as, in many cases, convectional heating was better than microwave dielectric heating. The MW (microwave) delivery mode had also an influence in the formation of by-products and, consequently, product yields. A tandem Sonogashira-cyclisation reaction was also accomplished using Pd(OAc)_2_/Xphos in the nanomicellar water environment.

## 1. Introduction

Amongst the plethora of methods to synthesize indoles, the cyclization reaction of 2-alkynylaniline derivatives is a significant approach to build 2-substituted- or 2,3-disubstituted-indoles [1,2,3]. One of the major benefits is the easy access to diverse 2-alkynylanilines through Sonogashira reaction from commercially available *ortho*-haloanilines and 1-alkynes [4,5]. Using strong bases or catalysis from transition metals are required to induce cyclization. Noble metals are generally employed [2], although catalytic (or almost stoichiometric) Cu derivatives have been employed [6,7], as other more affordable catalysts containing, for example, Hg [8], or Zn [9,10]. Metal catalysed cyclizative approaches to indoles have been recently reviewed [11,12]. Electrochemical cyclisation [13] or simple MW irradiation has been also reports as suitable technologies for cyclisation of 2-alkynyl-anilines [14].

Although the cyclization of 2-alkynylanilines has been dissected in most of its aspects, some issues are still unresolved. The need for alkynyl amines protected at N with electron-withdrawing groups (as Tf, Ts or Ns) [15,16,17], the use of ligands to stabilize the metal catalyst [14,15] or the employment of organic polar media as solvents [15,16] are some problems that should still be solved. Regarding the use of sustainable solvents, the use of copper catalysis in water have been recently reported for cyclisation of *N*-(2-ethynylphenyl)-sulfonamides to indoles [18,19].

Water is the environmentally benign solvent par excellence, often characterized by a different reactivity respect to organic solvents [20]. However, water is often not compatible with transition metal complexes and also low solubility of organic polar substrates hampers the success of many organic processes in aqueous media. Using surfactants appears as one of the simplest approaches to perform organic reactions in water [21,22,23]. The micellar environment enables the solubilization of organic lipophilic molecules in water behaving as a nanoreactor with a powerful influence on the reaction rate [24]. Recently a one-pot synthesis of indoles and pyrazoles have been reported under aqueous micellar catalysis [25]. The arylation of *N*-Boc-hydrazine was reported to occur in 3% TPGS-750-M in water in the presence of [*t*-BuBrettPhosPd-(allyl)]OTf. The further Fischer indole cyclisation of the arylhydrazine in the presence of ketones was carried out in the same pot under acid catalysis with good yields.

Following our interest in metal catalysed organic transformations in environmentally benign media [26,27], we became interested in studying the cyclisation of unprotected 2-alkynylaniline to 2-substituted indoles in a water/micellar environment. Starting from the unprotected aniline has the advantage to avoid the waste of reagents required for the protection and deprotection steps. The final indole could be also functionalized at the nitrogen with groups that may be not compatible with the cyclisation process.

## 2. Results and Discussion

Cyclisation of *o*-[2-(4-methoxy-3-tolyl)ethynyl]aniline (**1**) [28] was selected as the model reaction (Table 1). We explored different Pd catalyst/ligand combination to find the best conditions to have good conversion of the starting material into the cyclized product **2**. As surfactant, we opted for TPGS-750-M (Table 1), a designed surfactant already applied in several metal-catalyzed reactions [28,29,30]. Based on literature reports on analogous reaction, we decided to investigate MW dielectric heating conditions using Pd(II) catalyst. Using Pd(PPh_3_)_2_Cl_2_, Pd(MeCN)_2_Cl_2_ or Pd(OAc)_2_ complexed with Xphos gave totally unsatisfactory results with formation of small amount of indole and starting material recovered (entries 1–5 in Table 1). Even the use of oxime-derived palladacycles [31] gave a mixture of indole **2** and starting material (entry 6). After the discovery, by chance, that the ligand had no effect on the reaction yield (entry 8), we tried to increase reaction time and temperature to improve the amount of **2** formed. The major by-products identified were the reduced forms of the starting alkyne.

However, the final improvement occurred adding 1 eq of acetic acid that gave increased yields and moderate amount of unreacted starting material (entry 10 in Table 1). A minor change of reaction temperature and time gave the complete disappearance of the starting alkynylaniline **1** providing indole **2** in 70% isolated yield after column chromatography. Attempts to increase the solubility of Pd(II) in the micelles using a more lipophilic acid (lauric acid) or a better complexing agent 4-(diphenylphosphino)benzoic acid (DPBA), had no effect in the reaction yield (entries 16 and 17 in Table 1). Analogously, a stronger acid as TFA gave lower yield of indole **2** together with a series of by-products (entry 15). Using water alone or a solution of an ionic surfactant as 3% SDS, we observed incomplete conversion of the starting material (entries 21 and 22), demonstrating that the use of the nanomicelles formed from neutral surfactant TPGS-750-M in water, was decisive for the success of the reaction.

Finally we were pleased to find that, using 5% mol of Pd(OAc)_2_, cyclisation occurred heating at 100 °C for 10 min, giving 72% isolated yield of indole **2** (entry 18). Attempts to further reduce the amount of Pd gave incomplete conversion even after extended heating under MWs (entry 19).

Irradiation mode seemed to have an influence in the yield of **2**. Using the standard MW heating program that keeps the set temperature through magnetron on/off intervals, the crude reaction mixture obtained was characterized by several by-products and the yield of **2** was lower. Using the PowerMax™ technology, instead, using compressed gas to cool the reaction maintaining the temperature at 100 °C while the magnetron is on, gave a cleaner reaction mixture with less by-products resulting in higher isolated yield of indole **2** (entry 18). This last refined MW heating technology, however, did not prevent the formation of by-products as highlighted by the chromatographic analysis of the crude reaction mixture, probably due to the formation of hot-spot during irradiation [32]. A comparable result was observed with convectional heating of the reaction vial at 100 °C (external bath temperature) for 4 h, while longer reaction time produced a more complex mixture of by-products (entries 12 and 13 in Table 1).

**Scheme 1:**
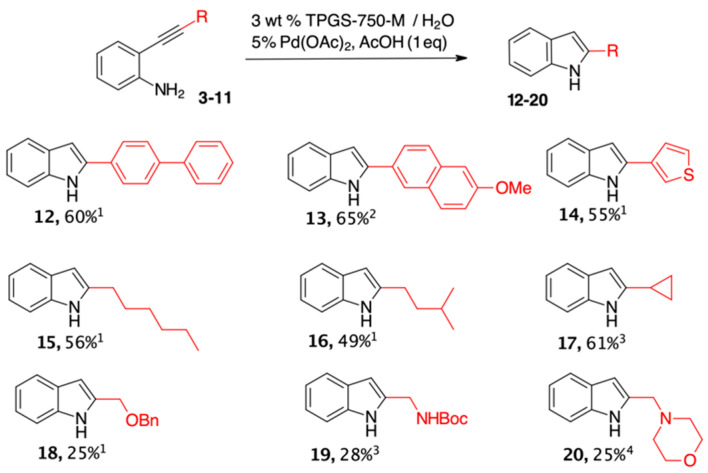
Scope of the reaction. ^1^ 80 °C, oil bath, 6 h. Yield obtained working under MW dieletric heating: (**12**, 35%), (**14**, 30%), (**15**, 27%), (**16**, 20%), (**18**, <10%). ^2^ MW (PowerMax on), see Supplementary Material (SM) ^3^ MW (PowerMax off), see SM. ^4^ Pd(OAc)_2_ (10%), see SM.

Thus, we explored lower temperature for longer time, and we were pleased to observe that heating at 80 °C for 6 h gave a slightly better yield (76%), mainly due to complete conversion of the starting material and formation of less by-products. Attempts to use MW at 80 °C, on the other hand, gave incomplete conversion of aniline **1** after 6 h of heating (data not shown). In conclusion we succeeded in find conditions for cyclisation of unprotected alkynyl-aniline **1** into indole **2** in water using either MW dielectric or convectional heating with a certain preference for the convectional mode. The reaction required the presence of AcOH, probably due to limited coordination of Pd to the free amine, assistance in triple bond activation and facilitated protonolysis of C-Pd bond. This trend did not change when differently substituted *o*-alkynyl-anilines [33] were submitted to cyclisation to access other 2-substitued indoles (Scheme 1). With-not-coordinating residues, cyclisation occurred with acceptable yields stirring for 6 h under convectional heating (products **12**–**16** in Scheme 1).

When more coordinative elements were inserted in the residue, cyclisation of the free amine was more difficult. As the presence of the acid environment was indispensable for cyclisation, the use of alkynes with heteroatoms or functional groups with acid-labile protections (e.g., TBDMS of THP) was not possible. With our procedure, the only indoles with heteroatoms present at the substituents in position 2, were compounds **18**–**20** in Scheme 1. 2-Benzyloxymethyl-indole **18** was isolated in low yield after a careful purification from different by-products. With morpholine as substituent (**20**), cyclisation was even harder as the corresponding alkynylaniline seemed very unstable under heating. After several attempts, product **20** was isolated after stirring the reaction mixture for 7 days at room temperature. Although the *tert*-butoxycarbonyl protective group is known to be thermally labile, indole **19** was obtained under MW dielectric heating. In this case MWs were better than oil bath, as convectional heating gave largely deprotection of the starting material. An analogous behavior was observed with cyclopropyl derivative **17** that was obtained in better yields under MWs. The MW heating mode had again an influence in the reaction success. The “harder” reaction conditions, using PowerMax on, gave crudes containing larger amounts of by-products respect to the MW based procedure that maintains constant the temperature switching off the magnetron for much of the reaction time.

Finally, we tried to carry out a one pot Sonogashira/indole cyclisation, by relying on the later insight in the Sonogashira copper free mechanism. A tandem Pd/Pd cycle linked, via a multistep transmetallation process, with Pd(II) species which takes the place of Cu(I), has been proposed, suggesting that some Pd(II) was always present in the medium at the end of the reaction [34].

**Scheme 2:**
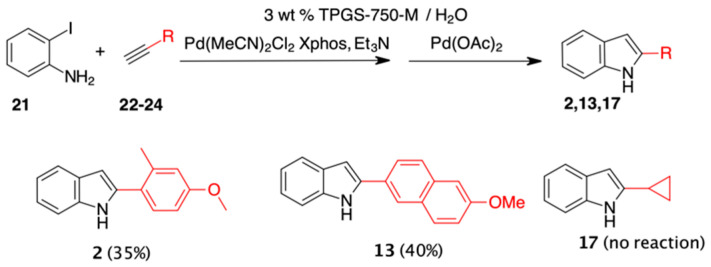
Tandem Sonogashira-indole cyclisation.

This hypothesis was explored starting from unprotected *o*-iodo-aniline **21** and alkynes **22**–**24** (Scheme 2). The first step was carried out in the presence of Et_3_N as the reaction requires an alkaline environment. When the starting aniline **21** was completely consumed, more Pd(OAc)_2_ was added and the vial heated at 100 °C under MWs. Following this procedure, compounds **2**, and **13** were obtained in acceptable yields while cyclization with alkyne **24** did not occur and only the Sonogashira product was recovered.

## 3. Materials and Methods

### 3.1. General Information

The reactions were carried out in a previously dried MW vial or Schlenk tubes. Product purification was carried out by flash column chromatography Merck silica gel 60, 0.040–0.063 mm (230–400 mesh) or using a 55 µm, 70 Å Strata^®^ SCX column from Phenomenex (Torrance, CA, USA) (phase: 2 g/12 mL strong cation exchange, SCX-SO_3_H). Merck (Darmstadt, Germany) aluminium-backed plates pre-coated with silica gel 60 (UV254) were used for analytical thin layer chromatography and were visualized by staining a solution of ninhydrin in EtOH.

^1^H-NMR (600 MHz) and ^13^C-NMR (JMOD) (150 MHz) spectra were recorded on an Avance NMR spectrometer (Bruker, Billerica, MA, USA). Deuterated chloroform, methanol and acetone were used as the solvent, and chemical shift values (δ) are reported in parts per million referred to the residual signals of this solvent (CDCl_3_ δ 7.26 for ^1^H and δ 77.16 for ^13^C; MeOD δ 3.31 for ^1^H and δ 49.00 for ^13^C; (CD_3_)_2_CO δ 2.05 for ^1^H and δ 205.87 and 30.70 for ^13^C). Data are represented as follows: chemical shift, multiplicity (s = singlet, d = doublet, t = triplet, q = quartet, m = multiplet or multiple resonances, bs = broad singlet), coupling constant (*J*) in Hertz and the integration. HPLC/MS analysis were performed with an Agilent 1260 Infinity II Preparative LC/MSD System Single Quadrupole (LC/MSD iQ, Agilent Technologies, Santa Clara, CA, USA) using an InfinityLab Poroshell 120 EC-C18 column (2.1 × 50 mm, 2.7 μm), flow 0.4 mL/min, MeCN/H_2_O gradient from 5/95 to 95/5 in 12 min. ES-MS analysis were performed with an Agilent 1100 series LC/MSD system, connected with a UV detector (254 nm), flow 0.4 mL/min, 95% MeOH, 5% H_2_O. Direct injection. ESI ionization, flow of the drying gas (N_2_) 9 mL/min, temperature 350 °C, atomizing pressure 40 PSI, fragmentation 70. Melting points were determined on an IA 9100 apparatus (Electrothermal, Staffordshire, UK). Elemental analyses were provided by an external supplier. Reactions carried out under MW dielectric heating were performed with a microwave oven (Discovery from CEM, Matthews, NC, USA) under monomode irradiation in a 10 mL sealed vials. The internal temperature was monitored through an internal IR sensor, and the maximal internal pressure monitored and maintained under the value of 150 psi. All reagents were used as purchased from commercial suppliers without further purification. Compounds **1** and **3**–**11** were prepared following literature procedures [28,35]. TPGS-750-M was used at higher concentration respect to the critical micellar concentration (CMC) [36].

### 3.2. Procedures

*2-(4-Methoxy-3-methylphenyl)-1H-indole* (**2**). General procedure, method A: In a Schlenk tube, a suspension of compound **1** (100 mg, 0.42 mmol) in TPGS-750-M 3 wt% in H_2_O (1 mL) was prepared under a nitrogen atmosphere. Acetic acid (24 µL, 0.42 mmol) and Pd(OAc)_2_ (5 mg, 0.02 mmol) were added sequentially. The reaction mixture was heated at 80 °C (external thermometer) in oil bath for 6 h, then diluted into EtOAc (5 mL). The organic layer was washed with H_2_O (2 × 5 mL) and brine (10 mL), dried over Na_2_SO_4_ and filtered. Removal of the solvent under reduce pressure gave a dark residue which was purified by silica gel flash chromatography or SCX column.

*2-(4-Methoxy-3-methylphenyl)-1H-indole* (**2**). General procedure, method B: A 10 mL MW tube was charged with a suspension of the compound **1** (100 mg, 0.42 mmol) in TPGS-750-M 3 wt% in H_2_O (1 mL). Acetic acid (24 µL, 0.42 mmol) and Pd(OAc)_2_ (5 mg, 0.02 mmol) were added sequentially under nitrogen atmosphere. The reaction mixture was submitted to microwave dielectric heating (MWs) for 10 min at 100 °C constantly cooling (ramp time 5 min; internal max pressure 250 psi; 150 W). Organic mixture was diluted into EtOAc (5 mL), the resulting phases were separated, and the organic layer was washed with H_2_O (2 × 5 mL), and brine (10 mL), dried over Na_2_SO_4_, filtered and the solvent was removed under vacuo. The dark residue was purified by flash chromatography on silica gel or SCX column. In alternative, the reaction mixture was submitted to microwave dielectric heating (MWs) for 3 cycles of 40 min at 80 °C (ramp time 1 min; internal max pressure 250 psi; power max 150 W) without constantly cooling.

*2-(4-Methoxy-3-methylphenyl)-1H-indole* (**2**) via Sonogashira/indole tandem cyclization reaction. A 10 mL MW tube was charged with iodoaniline (50 mg, 0.23 mmol), 4-ethynyl-1-methoxy-2-methylbenzene (50 μL, 0.34 mmol), triethylamine (80 μL, 0.58 mmol), a solution of TPGS-750-M 3 wt% in H_2_O (1 mL). Pd(CH_3_CN)_2_Cl_2_ (3 mg, 0.01 mmol) and Xphos (9 mg, 0.02 mmol) were added and the tube sealed. The mixture was submitted to microwave dielectric heating (MWs) for 30 min at 80 °C (max internal pressure 150 psi). Then Pd(OAc)_2_ (3 mg, 0.01 mmol) was added and the resulting mixture was heated under MW irradiations for further 10 min at 100 °C constantly cooling (PowerMax ON). The organic layer was diluted in EtOAc (5 mL) and washed with H_2_O (2 × 5 mL), dried over Na_2_SO_4_ and concentrated in vacuo. The residue was purified by silica gel flash chromatography (PE:EtOAc = 8:2) to afford **1** as a white solid (20 mg, 35%).

*2-(4-Methoxy-3-methylphenyl)-1H-indole* (**2**). Prepared from 2-((4-methoxy-3-methylphenyl)ethynyl)aniline in according with general procedure A, by heating at 80 °C (external thermometer) for 6 h in oil bath. Purified by silica gel flash chromatography (PE:EtOAc = 9:1). White solid; yield 76% (76 mg). Mp: 118–120 °C. MS-ESI: *m*/*z* 236 [M−H]^−^. ^1^H-NMR (600 MHz, CDCl_3_) δ 8.08 (s, 1H), 7.65 (d, *J* = 7.6 Hz, 1H), 7.40 (d, *J* = 6.9 Hz, 2H), 7.21 (t, *J* = 7.3 Hz, 1H), 7.15 (t, *J* = 7.3 Hz, 1H), 6.85 (dd, *J* = 11.8, 9.6 Hz, 2H), 6.55 (s, 1H), 3.86 (s, 3H), 2.49 (s, 3H). ^13^C-NMR (151 MHz, CDCl_3_) δ 159.29, 137.83, 137.46, 135.98, 130.28, 128.97, 125.38, 121.79, 120.35, 119.99, 116.41, 111.48, 110.66, 102.39, 55.33, 21.30. C_16_H_15_NO calcd C 80.98, H 6.37, N 5.90; O 6.74; found C 80.91, H 6.35, N 5.88.

*2-([1,1′-Biphenyl]-4-yl)-1H-indole* (**12**). Prepared from 2-([1,1′-biphenyl]-4-ylethynyl)aniline following general procedure A. Purified by SCX column eluting with MeOH. Pale yellow solid: yield 60% (60 mg). Mp: 281–283 °C. MS-ESI: *m*/*z* 268 [M−H]^−^. ^1^H-NMR (600 MHz, CDCl_3_) δ 8.35 (s, 1H), 7.76–7.62 (m, 6H), 7.53–7.35 (m, 5H), 7.21 (t, *J* = 7.6 Hz, 1H), 7.14 (t, *J* = 7.4 Hz, 1H), 6.89 (s, 1H). ^13^C-NMR (151 MHz, acetone-*d_6_*) δ 140.31, 139.78, 131.71, 128.90, 127.41, 127.30, 126.63, 125.51, 121.83, 120.20, 119.62, 111.11, 99.21. C_20_H_15_N calcd C 89.19, H 5.61, N 5.20; found C 89.24, H 5.64, N 5.23.

*2-(7-Methoxynaphthalen-2-yl)-1H-indole* (**13**). Prepared from 2-((7-methoxynaphthalen-2-yl)ethynyl)aniline following general procedure B. Purified by trituration with petroleum ether. Pale yellow solid; yield 65% (65 mg). Mp: 238–240 °C. MS-ESI: *m*/*z* 272 [M−H]^−^. ^1^H-NMR (600 MHz, acetone) δ 8.27 (s, 1H), 7.97 (dd, *J* = 8.5, 1.4 Hz, 1H), 7.87 (d, *J* = 8.5 Hz, 1H), 7.83 (d, *J* = 8.9, 1H), 7.58 (d, *J* = 7.7 Hz, 1H), 7.45 (d, *J* = 8.0 Hz, 1H), 7.31 (s, 1H), 7.18 (dd, *J* = 8.8, 2.3 Hz, 1H), 7.12 (t, *J* = 7.4 Hz, 1H), 7.03 (t, *J* = 7.3 Hz, 1H), 6.97 (s, 1H), 3.93 (s, 3H). ^13^C-NMR (151 MHz, acetone) δ 158.05, 138.08, 137.52, 134.20, 129.47, 129.19, 127.93, 127.43, 124.28, 122.97, 121.70, 120.14, 119.57, 119.25, 111.04, 105.92, 99.08, 54.80. C_19_H_15_NO calcd C 83.49, H 5.53; N 5.12; O 5.85; found C 83.53, H 5.50; N 5.08.

*2-(Thiophen-3-yl)-1H-indole* (**14**). Prepared from 2-(thiophen-3-ylethynyl)aniline by classical heating in oil bath at 80 °C (external thermometer) for 6 h. Purified by silica gel flash chromatography (PE:EtOAc = 9:1). White solid; yield 55% (55 mg). Mp: 210–213 °C. MS-ESI: *m*/*z* 198 [M−H]^−^. ^1^H-NMR (600 MHz, CDCl_3_) δ 8.27 (s, 1H), 7.62 (d, *J* = 7.7 Hz, 1H), 7.42 (s, 4H), 7.20 (t, *J* = 6.8 Hz, 1H), 7.14 (d, *J* = 6.8 Hz, 1H), 6.71 (s, 1H). ^13^C-NMR [37]. C_12_H_9_NS calcd C 72.33; H 4.55; N 7.03; S 16.09; found C 72.28; H 4.53; N 6.99.

*2-Hexyl-1H-indole* (**15**). Prepared from 2-(oct-1-yn-1-yl)aniline according to general procedure A, for 4 h. Purified by silica gel flash chromatography (PE:EtOAc = 9:1). Pale yellow oil; yield 56% (56 mg). MS-ESI: *m*/*z* 200 [M−H]^−1^H-NMR (600 MHz, CDCl_3_) δ 7.86 (s, 1H), 7.55 (d, *J* = 7.6 Hz, 1H), 7.31 (d, *J* = 7.8 Hz, 1H), 7.13 (t, *J* = 7.4 Hz, 1H), 7.09 (t, *J* = 7.4 Hz, 1H), 6.25 (s, 1H), 2.77 (t, *J* = 7.6 Hz, 2H), 1.75 (dd, *J* = 14.8, 7.3 Hz,25H), 1.49–1.32 (m, 6H), 0.93 (d, *J* = 6.7 Hz, 3H).^13^C-NMR [38]. HRMS (ESI) calcd for C_14_H_18_N [M−H]^−^ 200.1439; found 200.1438.

*2-Isopentyl-1H-indole* (**16**). Prepared from 2-(5-methylhex-1-yn-1-yl)aniline following general procedure A. Purified by SCX column eluting with MeOH. Waxy material; yield 49% (49 mg). MS-ESI: *m*/*z* 186 [M−H]^−^. ^1^H-NMR (600 MHz, MeOD) δ 7.39 (d, *J* = 7.8 Hz, 1H), 7.25 (dd, *J* = 8.0, 0.5 Hz, 1H), 7.01–6.94 (m, 1H), 6.94-6.86 (m, 1H), 6.10 (d, *J* = 0.5 Hz, 1H), 2.86–2.67 (m, 2H), 1.71–1.53 (m, 3H), 0.97 (d, *J* = 6.2 Hz, 6H). ^13^C-NMR (151 MHz, MeOD) δ 140.18, 136.45, 128.90, 119.74, 118.78, 118.29, 109.94, 97.84, 38.24, 27.46, 25.70, 21.45. HRMS (ESI) calcd for C_13_H_16_N [M−H]^−^ 186.1283; found 186.1284.

*2-Cyclopropyl-1H-indole* (**17**). Prepared from 2-(cyclopropylethynyl)aniline by heating under microwave irradiations at 80 °C for 3 cycles of 40 min, 150 W, 250 psi, PowerMax OFF. Purified by silica gel flash chromatography (PE:EtOAc = 9:1). Pale yellow solid; yield 61% (61 mg). Mp: 59–60 °C. MS-ESI: *m*/*z* 156 [M−H]^−^. ^1^H-NMR (600 MHz, CDCl_3_) δ 7.98 (s, 1H), 7.51 (d, *J* = 7.7 Hz, 1H), 7.29 (s, 1H), 7.13–7.08 (m, 2H), 6.16 (s, 1H), 1.12–0.56 (m, 5H). ^13^C-NMR [39] C_11_H_11_N calcd C 84.04, H 7.05, N 8.91; found C 83.97, H 7.03, N 8.88.

*2-((Benzyloxy)methyl)-1H-indole* (**18**). Prepared from 2-(3-(benzyloxy)prop-1-yn-1-yl)aniline following general procedure A. Purified by silica gel flash chromatography (PE:EtOAc = 6:4). Pale yellow oil; yield 25% (25 mg). MS-ESI: *m*/*z* 236 [M−H]^−^. ^1^H-NMR (600 MHz, MeOD) δ 7.49 (d, *J* = 7.8 Hz, 1H), 7.37–7.28 (m, 6H), 7.08 (t, *J* = 7.4 Hz, 1H), 6.98 (t, *J* = 7.4 Hz, 1H), 6.39 (s, 1H), 4.68 (s, 2H), 4.55 (s, 2H). ^13^C-NMR [40]. HRMS (ESI) calcd for C_16_H_14_NO [M−H]^−^ calcd 236.1075; found 236.1075.

*tert-Butyl((1H-indol-2-yl)methyl)carbamate* (**19**). Prepared from *tert*-butyl (3-(2-aminophenyl)prop-2-yn-1-yl)carbamate by heating under MW irradiations at 80 °C for 3 cycles of 20 min, 150 W, 250 psi. (In according with general procedure B). Purified by silica gel flash chromatography (PE:EtOAc = 8:2). White solid; yield 28% (28 mg). Mp 108–111 °C MS-ESI: *m*/*z* 245 [M−H]^−^. ^1^H-NMR (600 MHz, MeOD) δ 7.44 (d, *J* = 7.8 Hz, 1H), 7.30 (d, *J* = 8.0 Hz, 1H), 7.04 (t, *J* = 7.3 Hz, 1H), 6.95 (t, *J* = 7.3 Hz, 1H), 6.27 (s, 1H), 4.85 (s, 2H), 1.47 (s, 9H).^13^C-NMR (151 MHz, MeOD) δ 120.65, 119.42, 118.65, 110.40, 98.97, 37.52, 27.37. HRMS (ESI) calcd for C_14_H_17_N_2_O_2_ [M−H]^−^ 245.1290; found 245.1292.

*4-((1H-Indol-2-yl)methyl)morpholine* (**20**): a 25 mL bottom flask was charged with a suspension of 11 (90 mg, 0.42 mmol) in TPGS-750-M 3 wt% in H_2_O (1 mL); Pd(OAc)_2_ (10 mg, 0.04 mmol) were added sequentially under nitrogen atmosphere. The reaction mixture was stirred at room temperature for 7 days. The organic layer was diluted in CH_2_Cl_2_ (5 mL) and washed with H_2_O (2 × 5 mL), dried over Na_2_SO_4_ and concentrated in vacuo. The residue was purified by silica gel flash chromatography (CH_2_Cl_2_:MeOH = 85:15). Orange oil; yield 25% (23 mg). ^1^H-NMR (600 MHz, CDCl_3_) δ 8.87 (s, 1H), 7.68 (dd, *J* = 10.0, 8.9 Hz, 1H), 7.37 (d, *J* = 7.9 Hz, 1H), 7.18 (t, *J* = 7.4 Hz, 1H), 7.09 (t, *J* = 7.2 Hz, 1H), 6.41 (s, 1H), 3.78 (s, 6H), 2.59 (s, 4H). ^13^C-NMR [41]. The sample was contaminated with Ph_3_PO coming from the starting alkyne.

## 4. Conclusions

The TPGS-750-M/water environment was found to be a suitable medium to carry out cyclisation of *N*-unprotected *o*-alkynyl-aniline under Pd(OAc)_2_ catalysis. The reaction occurred without ligands but was very sensitive to the heating mode employed. Convectional heating resulted superior to MW dielectric heating as it allowed to work at lower temperature for longer times, thus reducing the number of by-products formed during the reaction. Importantly, when MWs were employed, the use of simultaneous cooling and continuous irradiation at variable power was more efficient for thermally stable substrates.

Although with low yields, we show also that a tandem Sonogashira-cyclisation reaction it is possible. Further optimization of the conditions is still required for application to larger scale processes, the procedure described here possess marked benefits in terms of atom economy and overall safety if compared with other previously reported methodologies.

## Figures and Tables

**Table 1:**
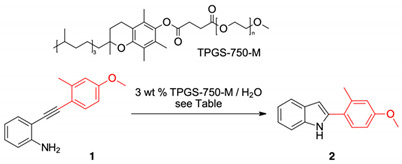
Optimization of reaction conditions.

Ent.	Metal Salts/Ligand ^1^	Additive	Type of Heating	Time (min)/ Temperature (T°) ^2^	Yield (2)	(1) Recovered
1	Pd(PPh_3_)_2_Cl_2_ (10 mol%)/XPhos (10 mol%)	--	MW	100 °C, 30 min ^3^	0%	80%
2	Pd(MeCN)_2_Cl_2_ (10 mol%)/XPhos (10 mol)	--	MW	100 °C, 30 min, then 150 °C 30 min ^3^	25%	60%
3	Pd(OAc)_2_ (10 mol%)/Xphos (10 mol%)	--	MW	100 °C, 30 min ^3^	25%	63%
4	Pd(OAc)_2_ (10 mol%)/Xphos (10 mol%)	--	MW	80 °C, 20 +10 min ^3^	25%	55%
5	Pd(OAc)_2_ (10 mol%)/PPh_3_ (10 mol%)	--	MW	80 °C, 20 +10 min ^3^	0%	82%
6	Pd-Oxime (10 mol)	--	MW	80 °C, 3 × 20 min ^3^	45%	30%
7	Pd(OAc)_2_ (10 mol%)		MW	80 °C, 3 × 20 min ^3^	35%	40%
8	Pd(OAc)_2_ (10 mol%)	--	MW	80 °C, 3 × 40 min ^3^	45%	25%
9	Pd(OAc)_2_ (10 mol%)	--	Oil bath	80 °C, 16 h	35%	30%
10	Pd(OAc)_2_ (10 mol%)	AcOH (1 eq)	MW	80 °C, 3 × 20 min ^3^	50%	10%
11	Pd(OAc)_2_ (10 mol%)	AcOH (1 eq)	MW	100 °C. 10 min	70%	--
12	Pd(OAc)_2_ (10 mol%)	AcOH (1 eq)	Oil bath	100 °C, 4 h	67%	--
13	Pd(OAc)_2_ (10 mol%)	AcOH (1 eq)	Oil bath	100 °C, 8 h	30%	--
14	Pd(OAc)_2_ (10 mol%)	AcOH (1 eq)	Oil bath	80 °C, 6 h	76%	--
15	Pd(OAc)_2_ (10 mol%)	TFA (1 eq)	MW	100 °C, 10 min	53%	--
16	Pd(OAc)_2_ (10 mol%)	C_11_H_23_COOH (15 mol%)	MW	100 °C, 10 min	40%	35%
17	Pd(OAc)_2_ (10 mol%)	DPBA (15 mol%)	MW	100 °C, 10 min	45%	30%
18	Pd(OAc)_2_ (5 mol%)	AcOH (1 eq))	MW	100 °C, 10 min ^4^	72%	--
19	Pd(OAc)_2_ (2 mol%)	AcOH (1 eq)	MW	100 °C, 10 min ^5^	43%	39%
20	Pd(OAc)_2_ (10 mol)	AcOH (1 eq)	MW	100 °C, 10 min ^6^	37%	40%
21	Pd(OAc)_2_ (10 mol%)	AcOH (1 eq)	MW	100 °C, 10 min ^7^	48%	35%
22	Pd(OAc)_2_ (10 mol%)	AcOH (1 eq)	MW	100 °C, 10 min ^8^	45%	35%

^1^ 0.1 eq of catalyst and ligand, unless otherwise reported, and 1 eq of AcOH. ^2^ For reactions under MW dielectric heating: 1 mmol of compound **1** was mixed with 1 mL of a degassed 3% solution of TGPS-750M in H_2_O followed by the catalyst. The vial was heated with the program keeping the temperature through continuous irradiation and contemporary cooling (PowerMax = on). Alternatively, the vial was immersed in an oil bath at a set temperature. ^3^ MW irradiation to maintain the settled temperature (PowerMax = off). ^4^ 0.05 eq of Pd(OAc)_2_ employed. ^5^ 0.02 eq of Pd(OAc)_2_ employed. ^6^ Reaction done in H_2_O with 3% of SDS. ^7^ Reaction done in pure H_2_O. ^8^ Reaction done in MeCN.

## Data Availability

Not applicable.

## Supplementary Materials

The following are available online. General experimental procedure and product characterization, ^1^H and ^13^C-NMR spectra Figures S1–S15 and HPLC spectrum Figure S16.

## Sample Availability

Samples of compounds **2**, **12**–**20** are available from authors.
