# Supplementary material for: Synthesis of 2-Substitued Indoles via Pd-Catalysed Cyclization in an Aqueous Micellar Medium
Source: Molecules. 2021 Jun 26;26(13):3917. doi: 10.3390/molecules26133917 (PMC8271580; doi:10.3390/molecules26133917)

Synthesis of 2-Substitued Indoles via Pd-Catalyzed Cyclization in Aqueous Micellar Medium

Sofia Siciliano, Elena Cini, Maurizio Taddei* and Giorgia Vinciarelli

Dipartimento di Biotecnologie, Chimica e Farmacia, Università degli Studi di Siena, Via A. Moro 2, 53100 Siena, Italy

Supporting information

*2-(4-Methoxy-3-methylphenyl)-1*H*-indole* (**2**) (Figure S1-S2)

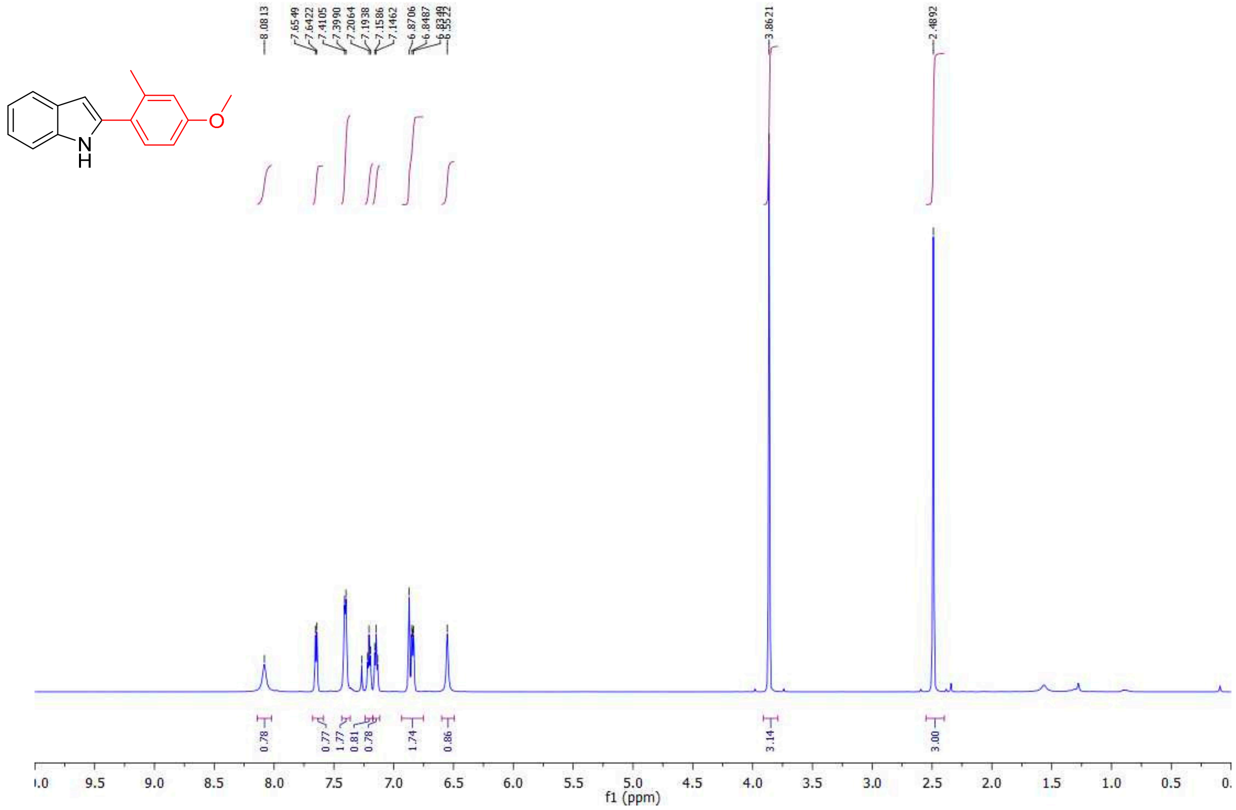

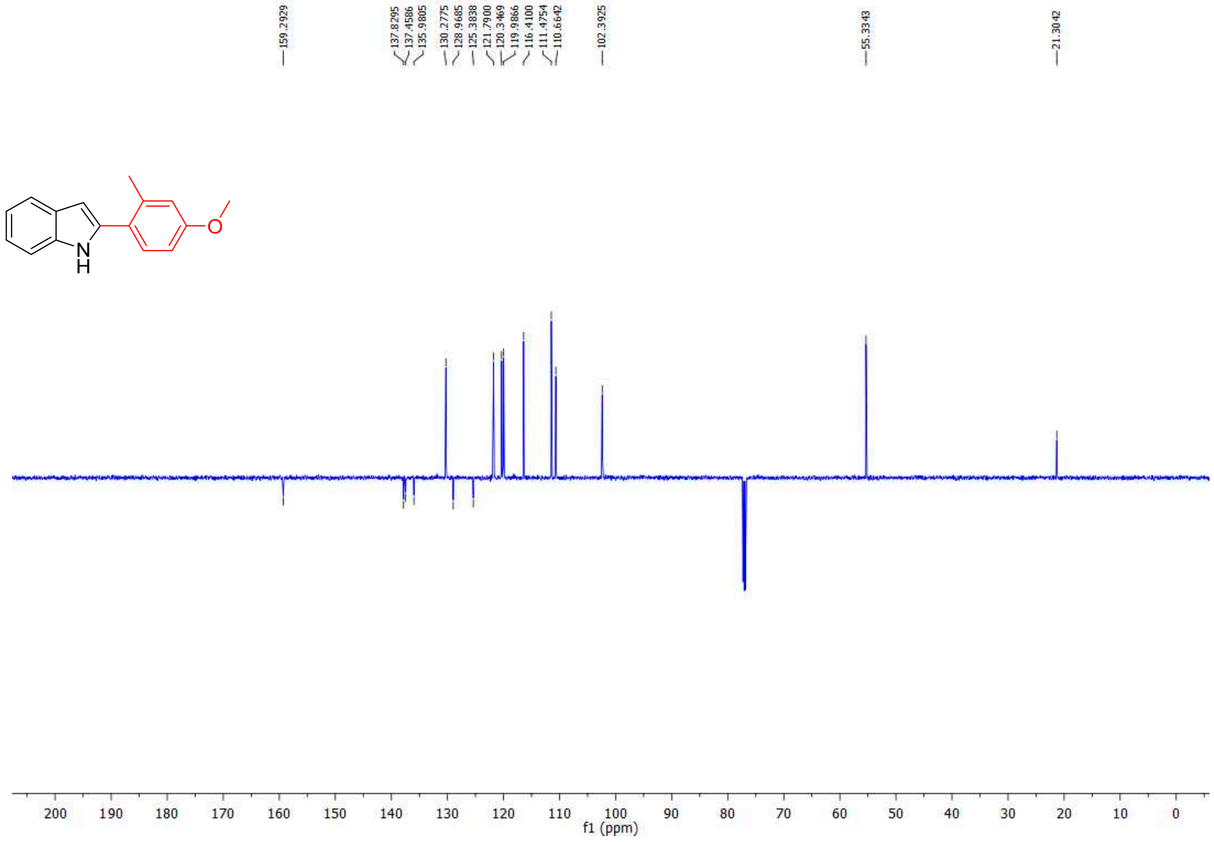

*2-([1,1'-Biphenyl]-4-yl)-1*H*-indole* (**12**) (Figure S3-S4)

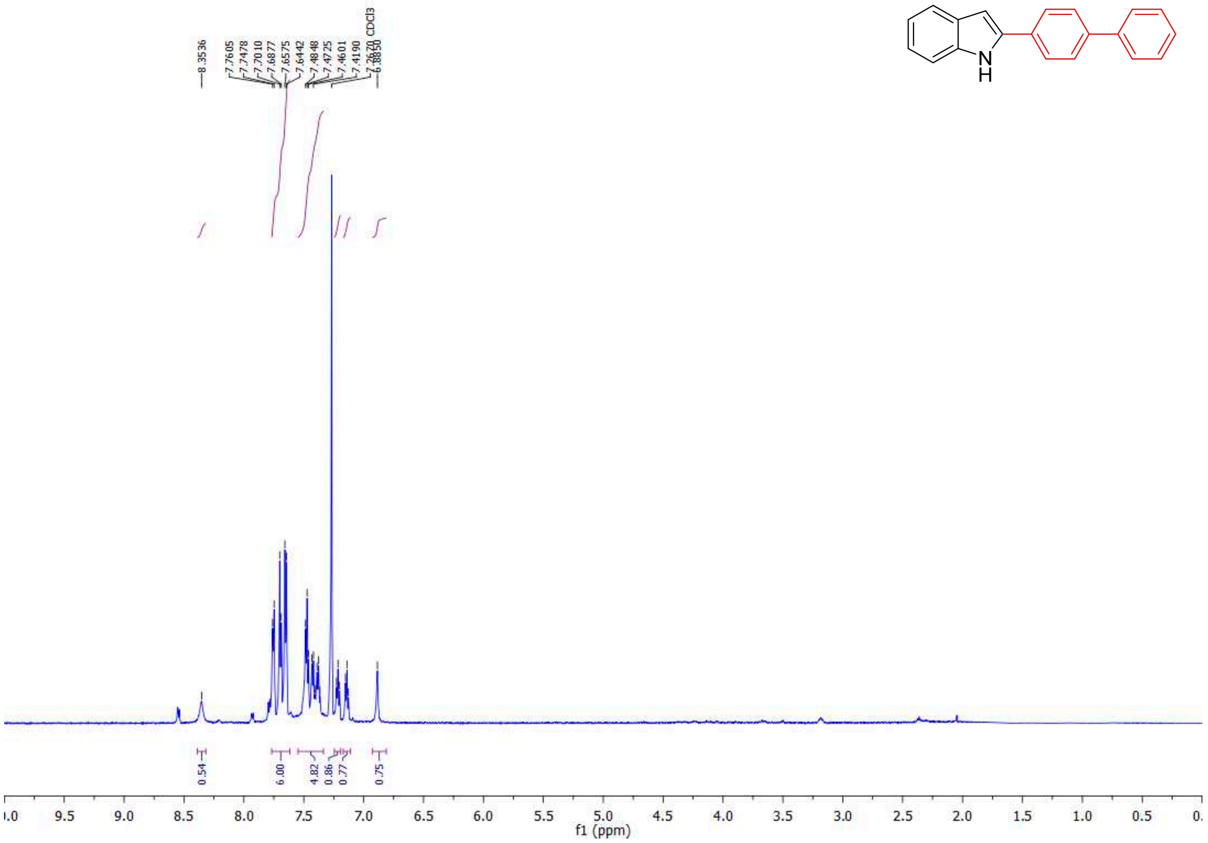

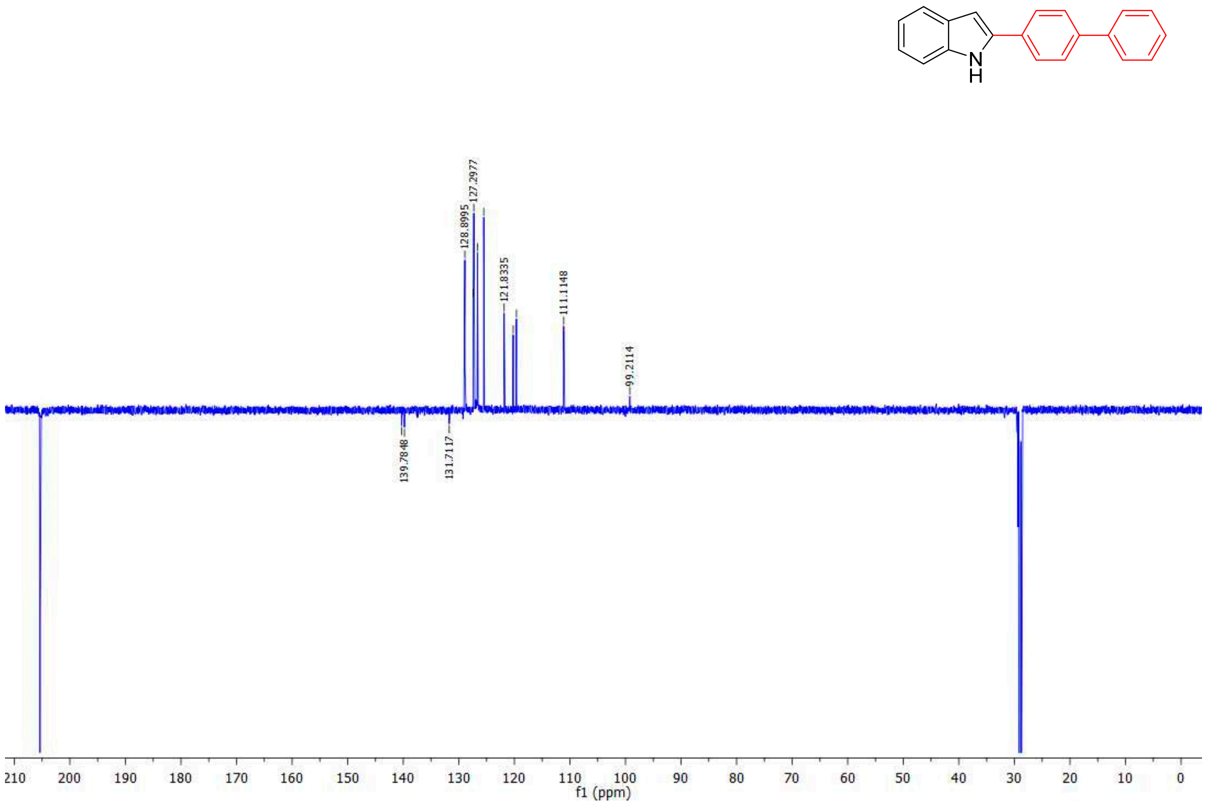

*2-(7-Methoxynaphthalen-2-yl)-1*H*-indole* (**13**) (Figure S5-S6)

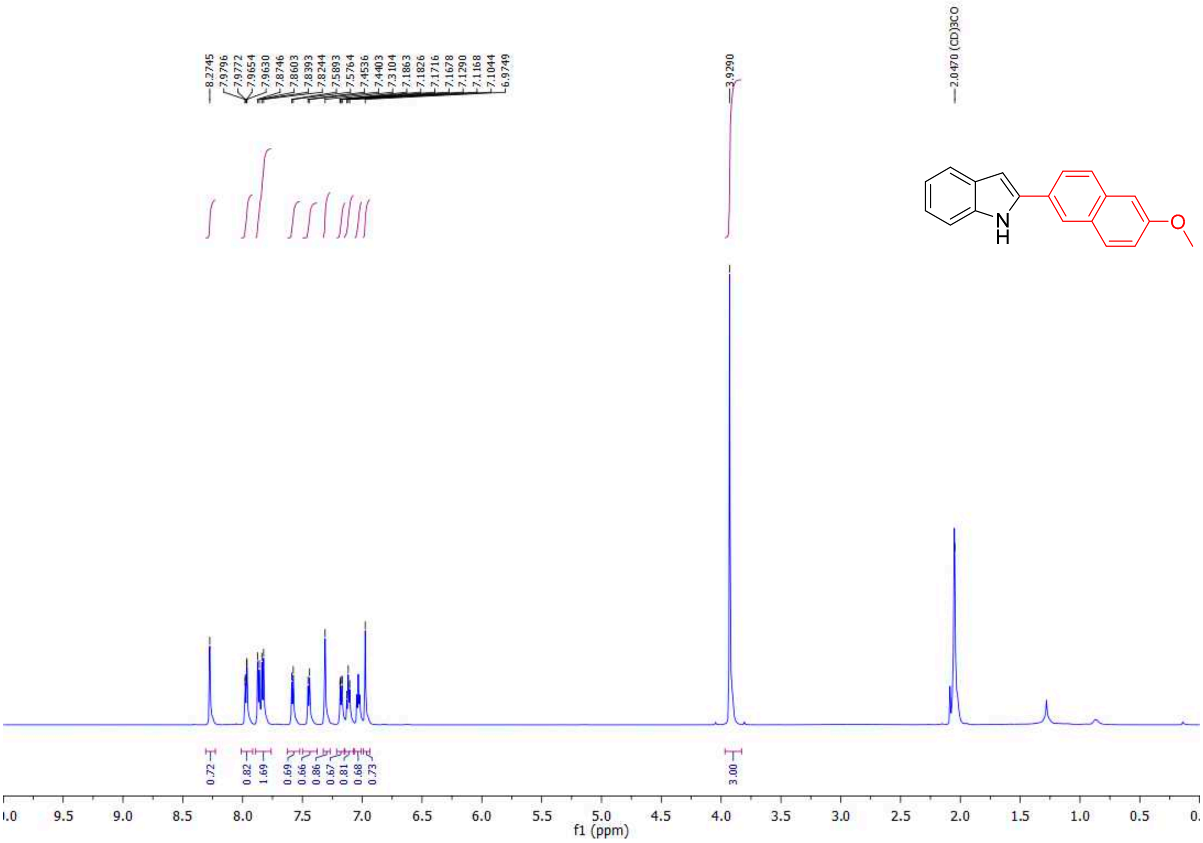

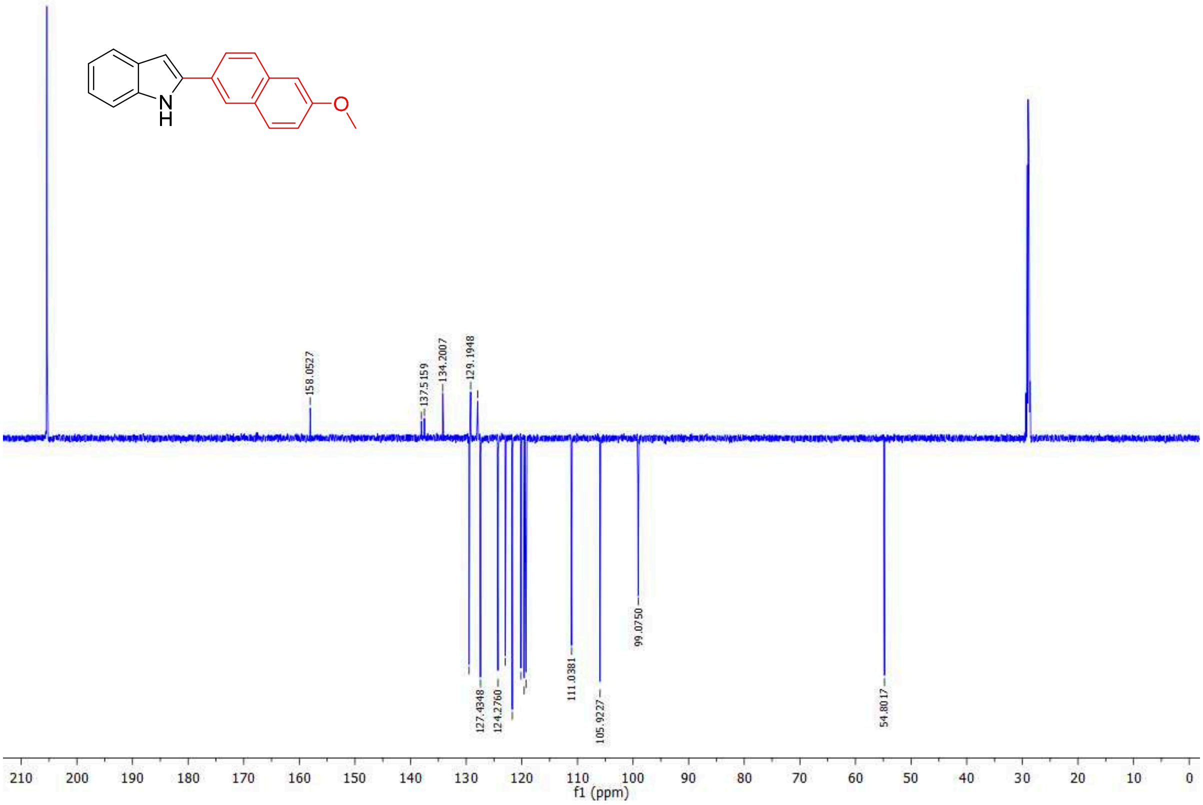

*2-(Thiophen-3-yl)-1*H-*indole* (**14**) (Figure S7)

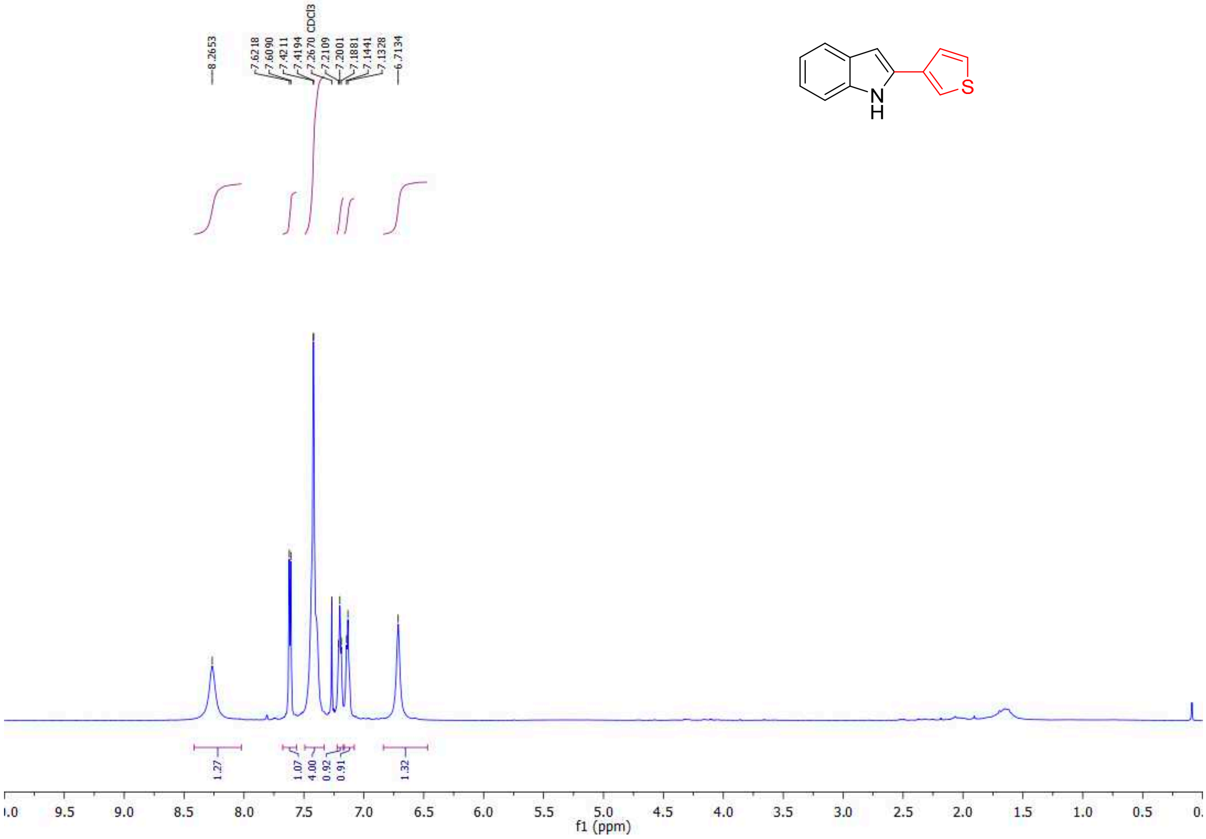

*2-Hexyl-1*H-*indole* (**15**) (Figure S8)

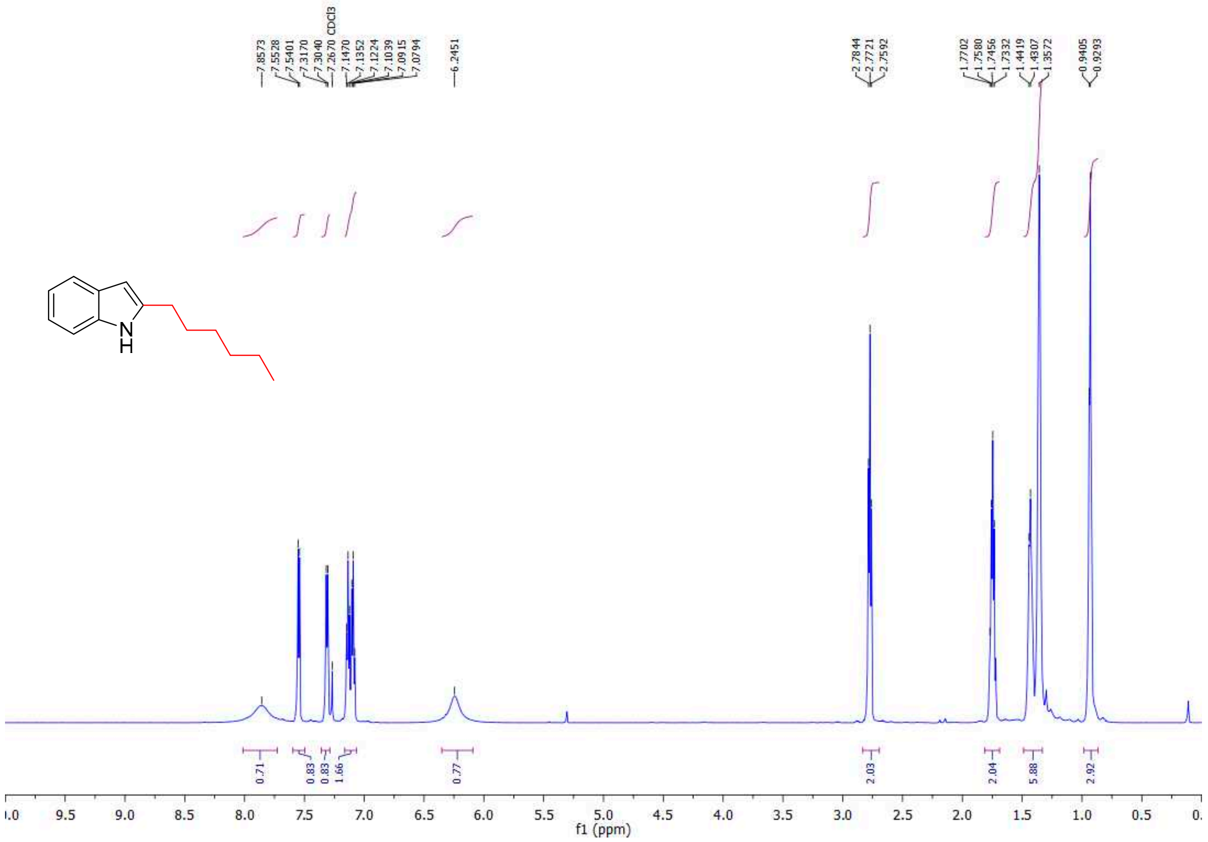

*2-Isopentyl-1*H-*indole* (**16**) (Figure S9-S10)

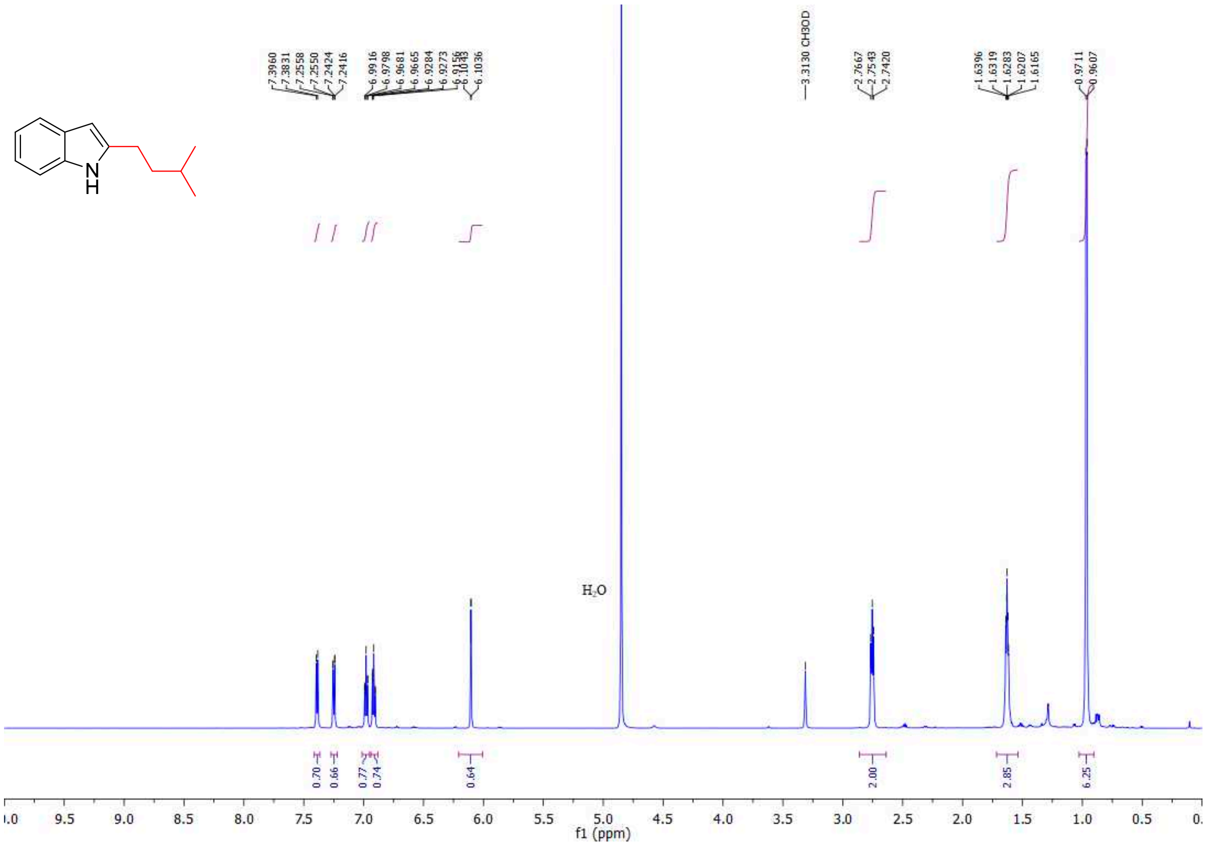

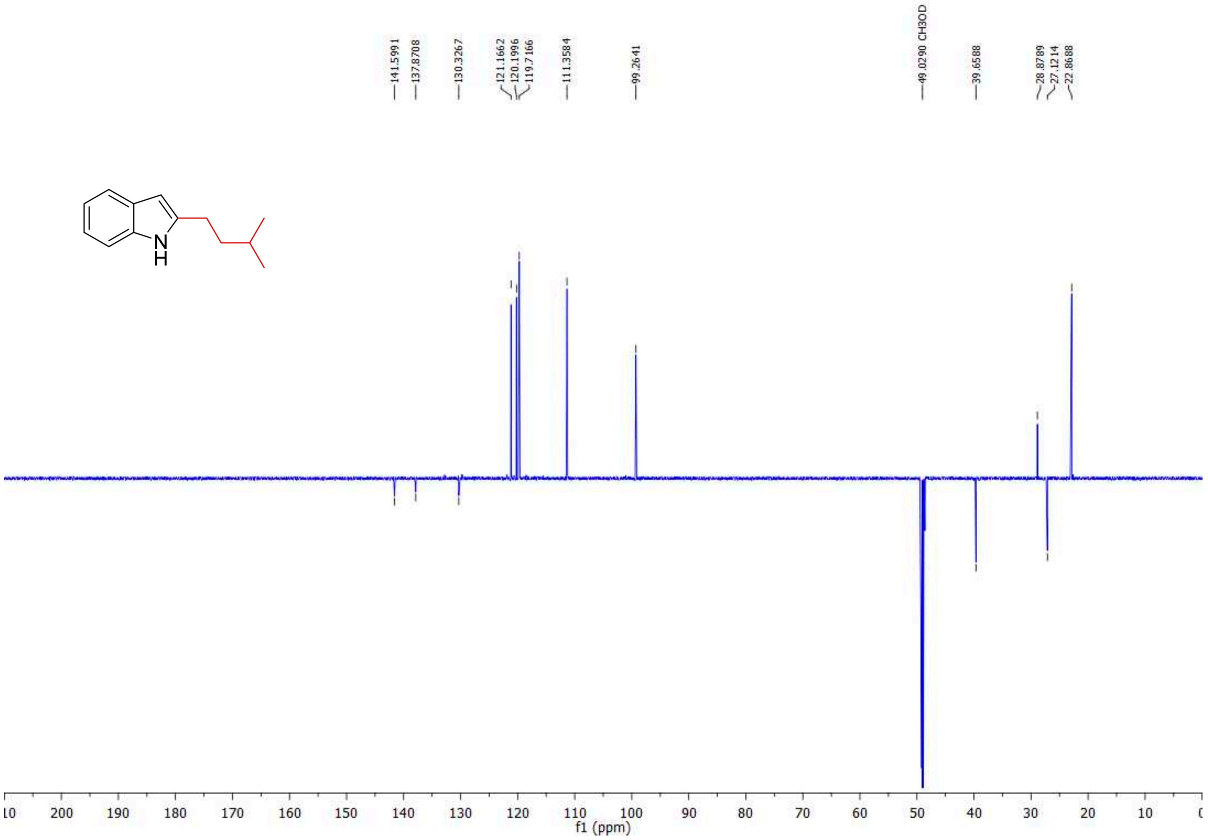

*2-Cyclopropyl-1*H-*indole* (**17**) (Figure S11)

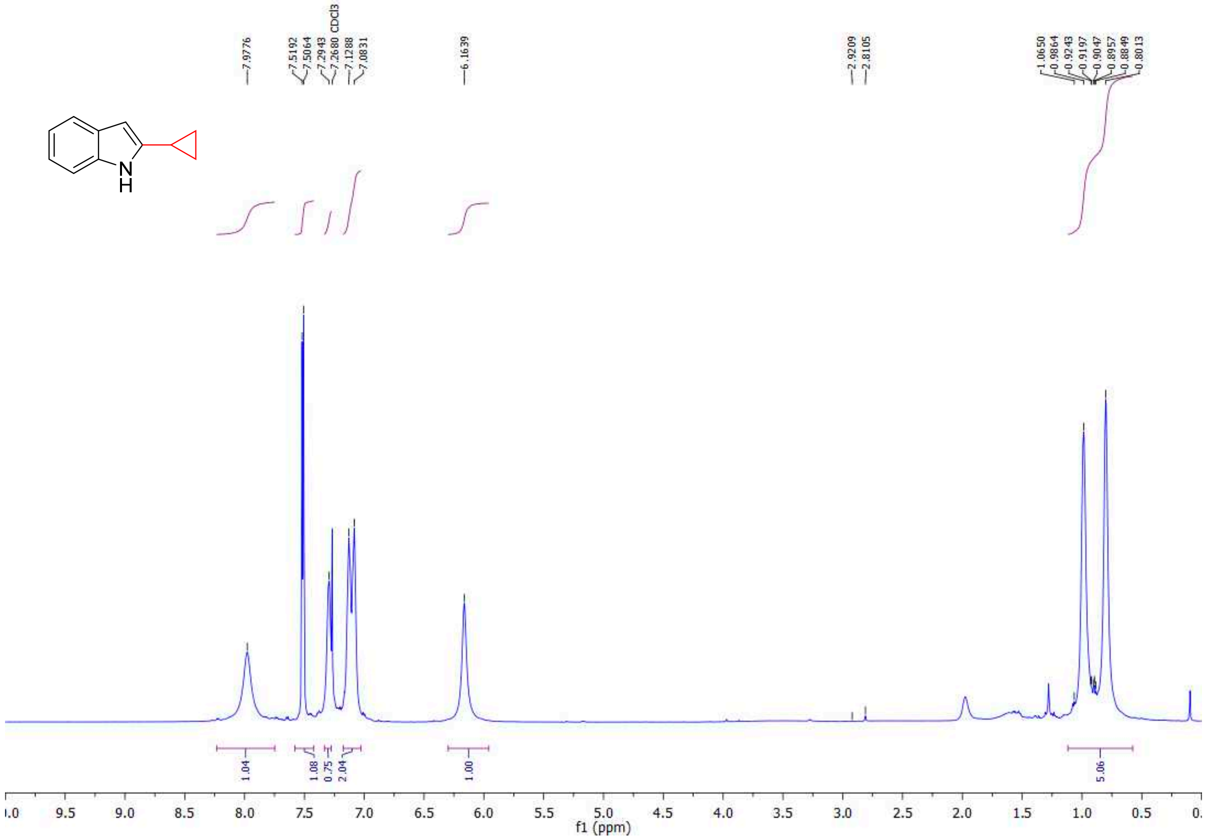

*2-((Benzyloxy)methyl)-1*H-*indole* (**18**) (Figure S12)

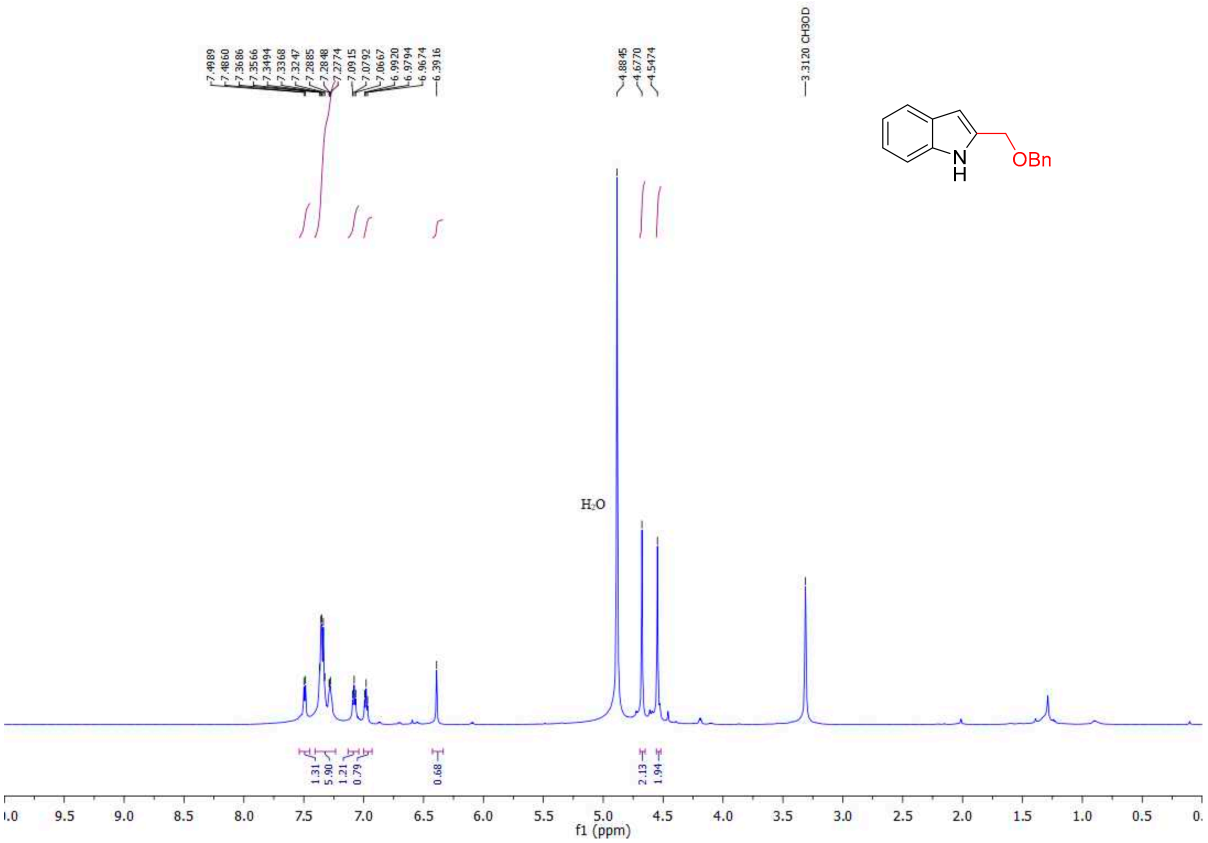

*tert-Butyl ((1*H*-indol-2-yl)methyl)carbamate* (**19**) (Figure S13-S14)

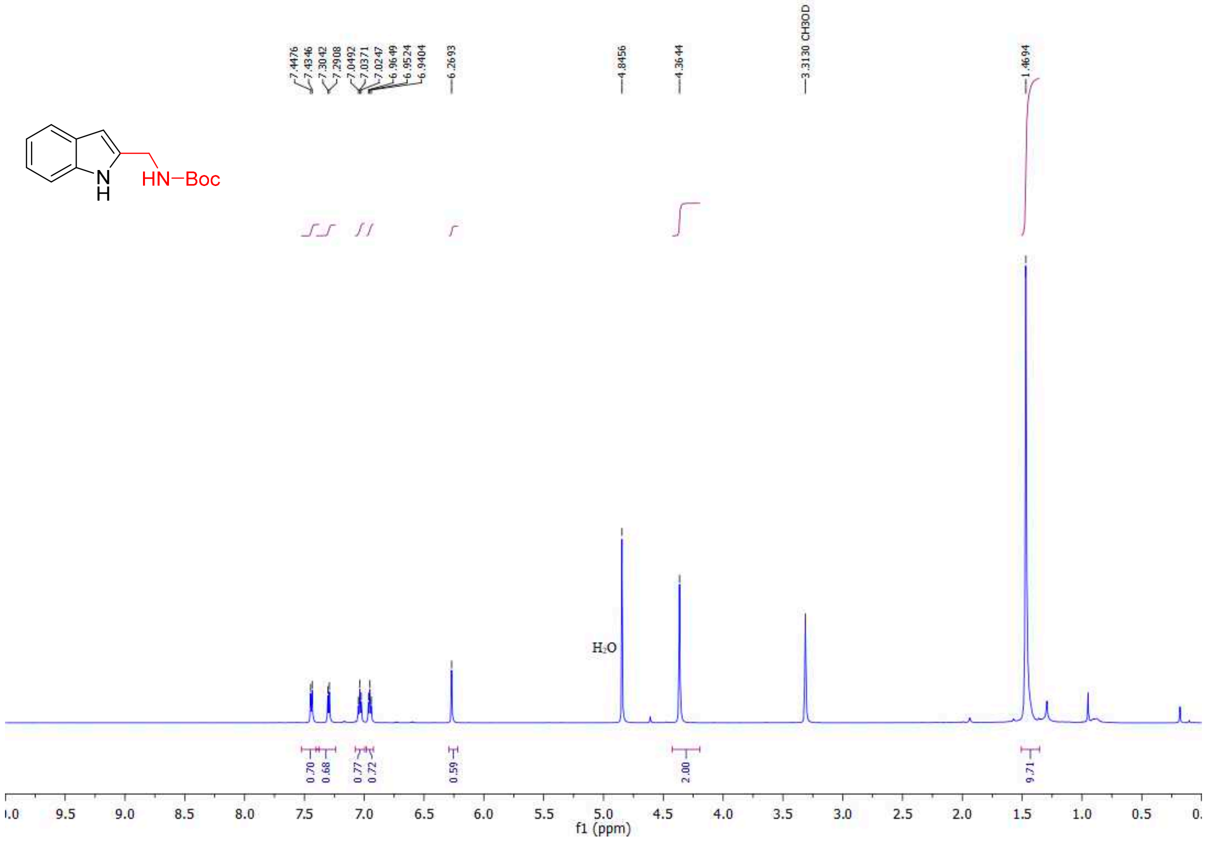

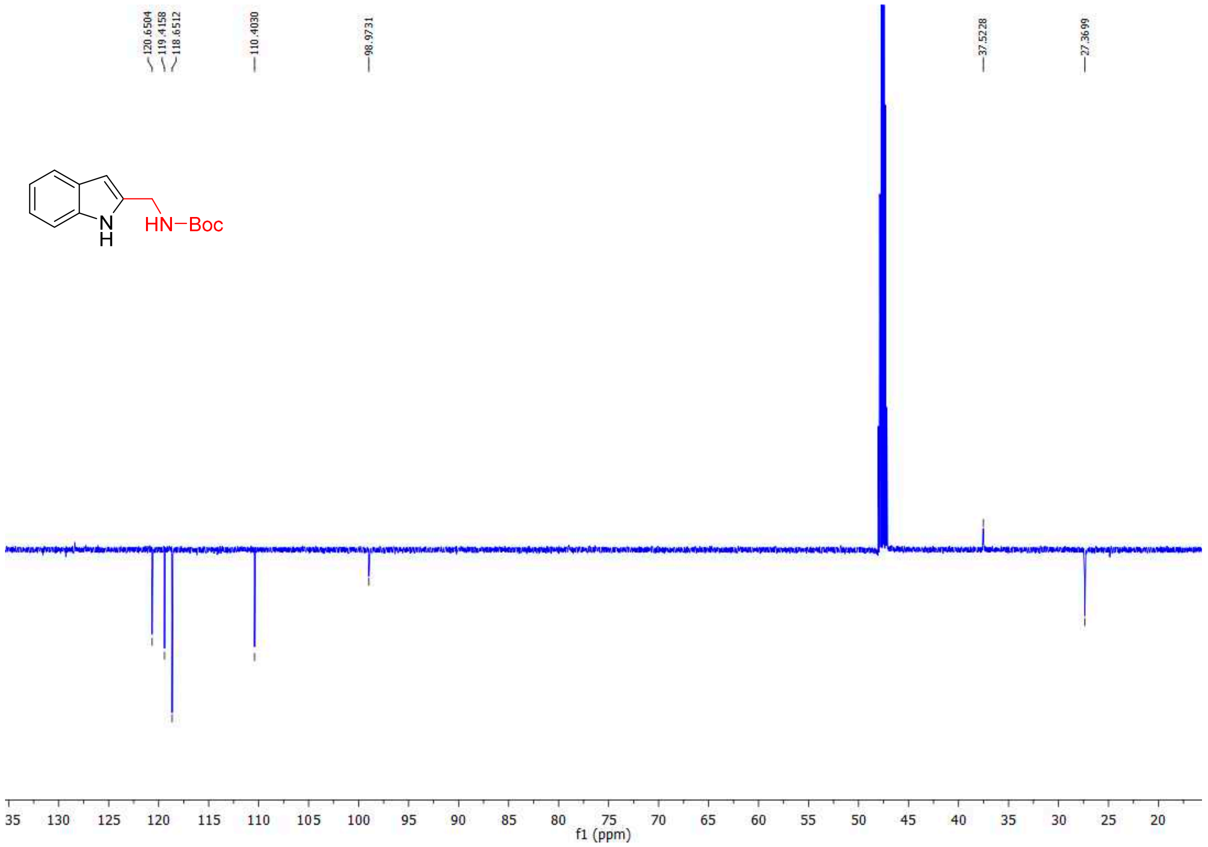

*4-((1*H*-indol-2-yl)methyl)morpholine* (**20**) (Figure S15-S16)

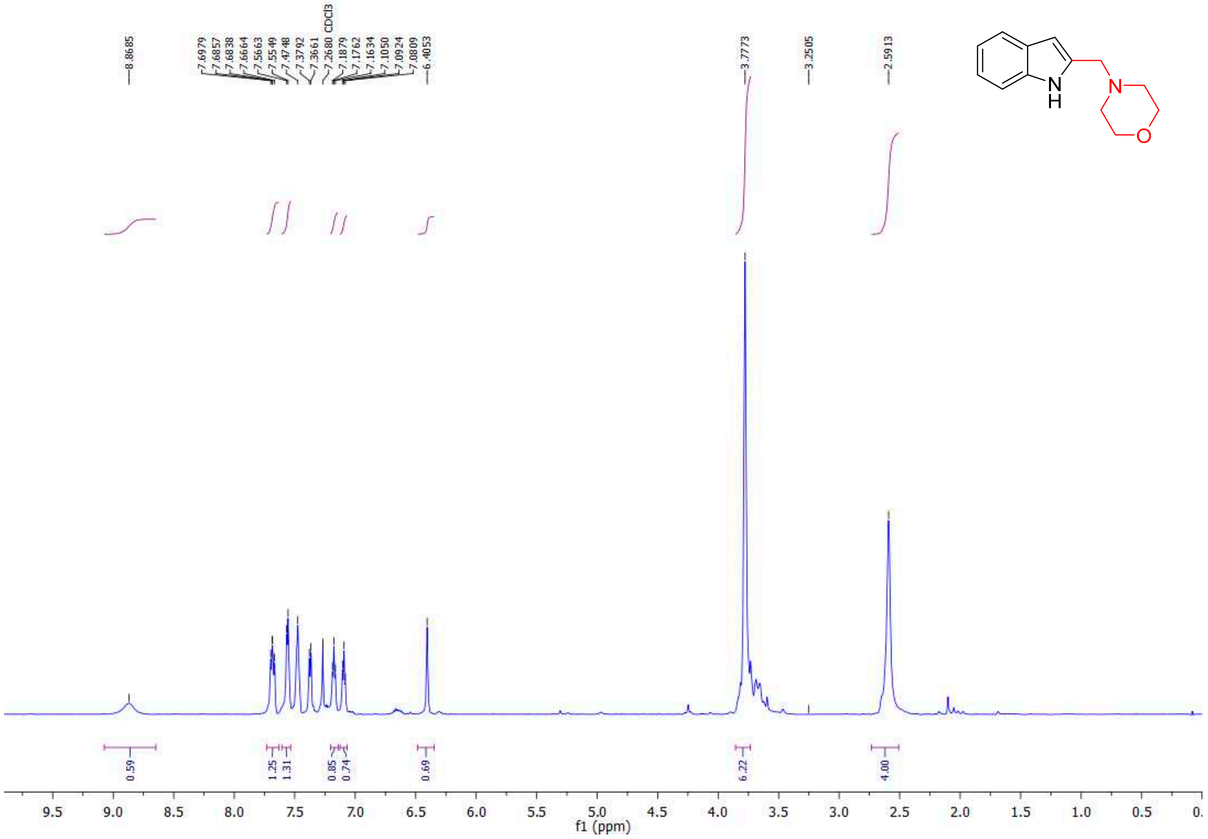

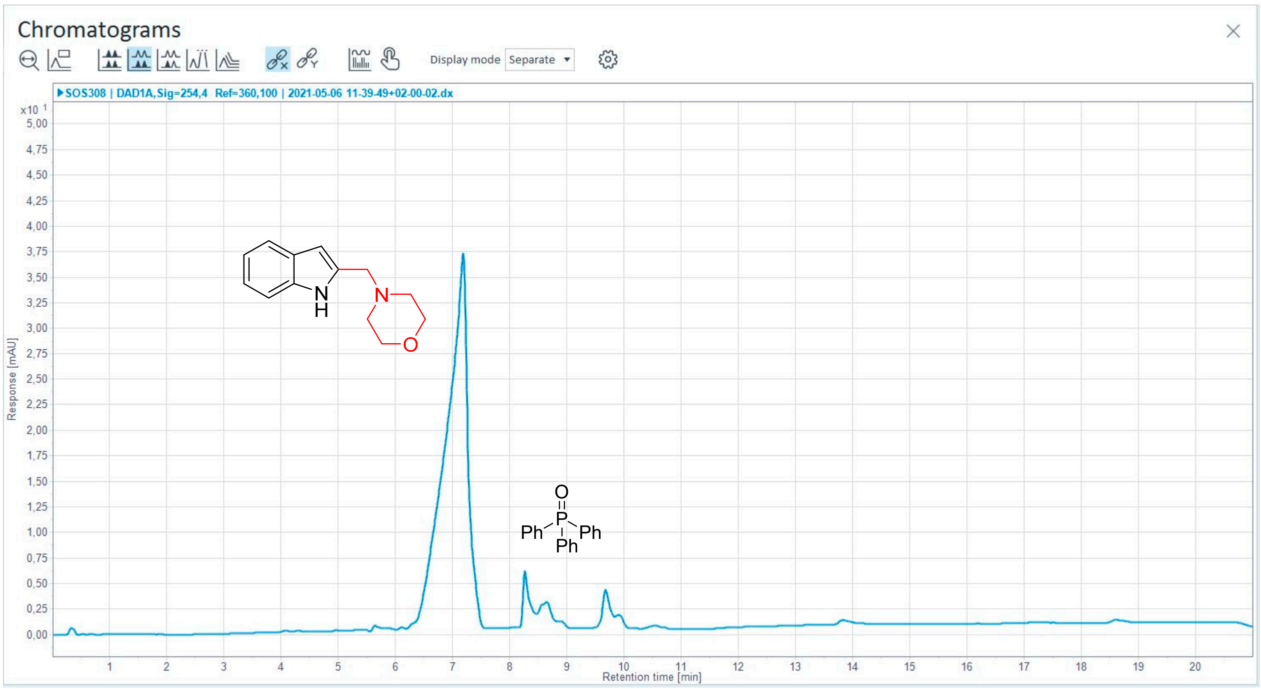

Supplement: Supplementary file 1 [file molecules-26-03917-s001.zip › molecules-1244673-supplementary.pdf]
